# Lifetime performance traits of Holstein cows: implications of first-lactation milk yield and culling causes

**DOI:** 10.1007/s11250-025-04670-7

**Published:** 2025-10-27

**Authors:** Nadia H. Fahim, Mohamed A. M. Ibrahim, Rabie R. Sadek

**Affiliations:** Animal Production Department, Faculty of Agriculture, Cairo University, Giza, Egypt

**Keywords:** Holstein, Culling, First-lactation milk, Lifetime performance

## Abstract

The study examined the impacts of first-lactation milk yield and culling causes on lifetime performance traits in Holstein cows culled after their second to sixth parities. Complete lactations from 12,209 records belonging to 4522 culled cows were included in the study. Reproductive problems, mastitis, metabolic disorders, lameness, endemic diseases, and respiratory diseases represented culling causes. The resulting means were 371 ± 2.0 days for days in milk 30,223 ± 374 kg for lifetime milk yield, 13.5 ± 0.4 kg for milk per day of cow age, 38 ± 0.4 months for productive life, 68 ± 0.4 months for longevity, 3.6 ± 0.05 for services per conception, and 437 ± 3.4 days for calving interval. The first-lactation milk yield was associated with lifetime milk yield, milk/day of age, no-services/conceptions, and calving intervals. Cows culled for reproductive problems exhibited the greatest longevity, whereas those culled for endemic diseases had the shortest. Cows culled for lameness achieved the highest lifetime milk yield, while cows culled for metabolic disorders showed the highest milk yield per day of cow age. Furthermore, reproductive problems, metabolic diseases, or mastitis caused a higher number of no-services/conceptions and calving intervals compared with the other culling causes. In conclusion, higher first lactation milk yield was associated with higher productivity and lower reproductive performance. Moreover, the lifetime performance of culled cows differed according to the reason for culling. Dairy management programs should address reasons for culling in terms of their interrelationships, as one reason may cause or promote the appearance of another.

## Introduction

Culling is one of the main factors that directly impacts milk production revenues. Culling decisions are typically made for cows with a deficiency in one or more performance traits closely linked to milk production. Lifetime performance summarizes the characteristics of the cows during their time in the herd. The most crucial lifetime characteristics are longevity and productive life (Jovanovac et al. 2013). Productive life (PL) is the interval between the first calving and the culling, while longevity is the duration from cow birth to culling. The extended lifespan of dairy cows increases the chances of voluntary culling, providing additional options for heifer sales, which benefit herd income (De Vries and Marcondes 2020). Cows that stay in the herd for five lactations can save 74% on heifer replacement costs compared to cows that are culled after their first lactation. PL is associated with the performance features; long PL increases the cumulative milk and the number of offspring (Caetano et al. 2017; Krpálková et al. 2017). A lengthy PL should be paired with a high daily milk to achieve high returns (Jenko et al. 2015). Reproductive characteristics are another critical performance factor that significantly impacts cow profitability; the rise of services per conception (NSPC) and calving intervals (CI) is linked with high insemination costs, a decline in lifetime milk (LTMY), a high culling percentage, and a poor selection intensity (Togashi and Lin 2008). Additional performance factors are lifetime productivity, average lifetime productivity over a specific time, and number of lactations (Sawa and Krȩzel-Czopek 2009; Heinrichs and Heinrichs 2011).

Investigating the factors influencing lifetime performance attributes may offer chances to enhance these features through breeding program design. Cows’ reproductive and productive traits in early lactations determine how they will function (Tamboli et al. 2021). Because the first lactation milk indicates the potential for production in the subsequent lactations, they could be predicted based on that milk yield. It is crucial for culling and selection (Marinov et al. 2020). There may be a connection between specific culling factors and dairy cows’ lifetime performance (Adamczyk et al. 2017). Studies on the relationship between definite performance traits and the causes behind dairy cow culling are required (Sawa et al. 2019). There was general research on the influence of first-lactation milk on dairy cows’ lifetime performance, but no studies were conducted on culled cows. Moreover, the effects of culling causes on lifetime performance attributes in culled cows have also not been investigated. As a result, this study aimed to investigate the impacts of first-lactation milk and various culling reasons on the lifetime performance characteristics of Holstein cows that had been culled over 10 years.

## Materials and methods

### Ethics approval

The Ethics Committee of Cairo University Institutional Animal Care and Use Committee (CU-IACUC) approved the study with the protocol code: CUIIRF224.

### Source of data

The data used in this study belonged to a large dairy farm, 80 kilometers northwest of Cairo and west of the Cairo-Alexandria Desert Road, Egypt. The farm was located 75 meters above sea level, at a latitude of 30.14° and a longitude of 30.33°. The data were collected from Dairy Comp305 (Valley Ag Software, Tulare, CA). The study used 12,209 complete lactation records from 4,522 second to sixth parity cows culled between 2008 and 2019. Data were categorized by culling causes, first-lactation milk, year, and calving season (Table 1).

**Table 1:** Data categories of culling causes, first-lactation milk, year, and season of calving for 4522 Holstein dairy cows culled during 2008–2019

Factor	Category		Numbers	%
Culling cause				
	Low milk yield		391	8.6
	Mastitis		1085	23.9
	Reproductive problems^1^		854	18.9
	Metabolic disorders^2^		619	13.7
	Lameness		518	11.5
	Endemic diseases^3^		496	11.0
	Respiratory diseases		198	4.4
	Unknown		361	8.0
First-lactation milk production				
	< 9,000 kg		1496	33.2
	9,000–12,000 kg		1747	38.6
	> 12,000 kg		1279	28.2
Year of calving				
	2008–2011		1026	22.6
	2012–2015		2770	61.3
	2016–2019		726	16.1
Season of calving				
	Winter		1191	26.3
	Spring		1078	23.9
	Summer		1044	23.1
	Autumn		1209	26.7

^1^ Reproductive problems involved dystocia, metritis, pregnancy toxemia, uterine prolapse, and uterine adhesion

^2^ Metabolic disorders include ketosis, hypocalcemia, displaced abomasum, and other less common metabolic disorders recorded in farm health records

^3^ Endemic diseases, represented by foot &mouth disease, bovine ephemeral fever, and lumpy skin disease

### Animals’ management

Under an open yard system covered with corrugated metal sheets, cows were housed in free-stall barns. Sand and earth made up the floors of the yards. Total mixed ration (TMR) was provided throughout the year. The rations were supplied thrice daily based on the cow’s body weight and milk production level. All day, freshwater was available. For the first time, heifers weighing approximately 370 kg were artificially inseminated. Machine milking was conducted three times a day, every eight hours. Two months in advance of the expected dates of calving, cows were dried off. A cooling system was installed at the farm. Every cow was raised under identical circumstances and with identical management.

### Studied traits

- Number of lactations (NL): the parities the cow gave during her lifespan.
- Days in milk (DIM, day): the time the cow was milked throughout her parity.
- Lifetime total milk yield (LTMY, kg): the milk produced by the cow while in the herd. It was estimated by adding the milk the cow produced over her lactations.
- Lifetime daily milk yield (LDMY, kg): milk yield daily for the age of a cow. LTMY divided by longevity in days was used to calculate it.
- Productive life (PL, month): the time passed between the cow’s first calving date and its last milk record.
- Longevity: months between the cow’s birthdate and culling date (month).
- Number of services per conception (NSPC): the services needed for every cow’s conception.
- Days open (DO, day): the time between calving and conception.
- Calving interval (CI, day): days between two consecutive calvings.

### Statistical analysis

Data were analyzed by XLSTAT version 2024.1. The general linear model (GLM) was applied to examine the influences of first-lactation milk and culling causes on the productive and reproductive characters of Holstein cows. Analysis of Variance (ANOVA) was used to inspect the significance of the fixed effects. If significant differences existed (*p* < 0.05), pair-wise comparisons among levels of each fixed factor were conducted by Tukey’s Honest Significant Difference (HSD) test to permit control for Type I error for multiple comparisons. When comparisons between two groups were made, independent samples t-tests were employed.

## Results

### Impacts of first-lactation milk yield (FMY) on lifetime performance traits

All studied traits were influenced by FMY (*p* < 0.0001). Table 2 displayed that cows with larger milk yields had fewer NL but higher LTMY and LDMY. Moreover, cows that produced FMY > 12,000 kg scored the greatest PL and longevity. Table 3 showed that NSPC increased with the rise in FMY. It doubled in the cows that produced FMY > 12,000 kg compared with those that produced FMY < 9,000 kg. Correspondingly, DO and CI elongated with the increase in FMY.

**Table 2:** Effects of first-lactation milk yield on productive traits in holstein dairy cows culled during 2008–2019

Productive traits	First-lactation milk production (kg)			P-value
	< 9,000	9,000–12,000	> 12,000	
NL	2.9^c^ ± 0.03	2.8^b^ ± 0.02	2.6^a^ ± 0.03	< 0.0001
DIM (day)	329^a^ ± 2.0	358^b^ ± 1.8	427^c^ ± 2.4	< 0.0001
LTMY (kg)	26.998^a^ ± 369	29.815^b^ ± 337	33.859^c^ ± 418	< 0.0001
LDMY (kg)	12.2^a^ ± 0.05	13.5^b^ ± 0.04	14.9^c^ ± 0.06	< 0.0001
PL (month)	36^a^ ± 0.4	37^a^ ± 0.04	41^b^ ± 0.5	< 0.0001
Longevity (month)	66^a^ ± 0.4	67^a^ ± 0.04	72^b^ ± 0.4	< 0.0001

NL: number of lactations, DIM: days in milk, LTMY: lifetime total milk yield, LDMY: lifetime daily milk yield, and PL: productive life. Means within rows followed by different letters are significantly different (*p* < 0.05)

**Table 3:** Effects of first-lactation milk yield on reproductive traits in holstein dairy cows culled during 2008–2019

Reproductive traits	First-lactation milk production (kg)			P-value
	< 9,000	9,000–12,000	> 12,000	
NSPC	2.5 ^a^ ±0.05	3.4 ^b^ ±0.04	5.2 ^c^ ±0.05	< 0.0001
DO (days)	124 ^a^ ±2.1	154 ^b^ ±1.8	228 ^c^ ±2.3	< 0.0001
CI (day)	390 ^a^ ±2.1	422 ^b^ ±1.8	501 ^c^ ±2.3	< 0.0001

NSPC: number of services/conceptions, DO: days open, and CI: calving interval. Means within rows followed by different letters are significantly different (*p* < 0.05)

### Impacts of culling causes on lifetime performance traits

#### Productive traits

**Fig. 1:**
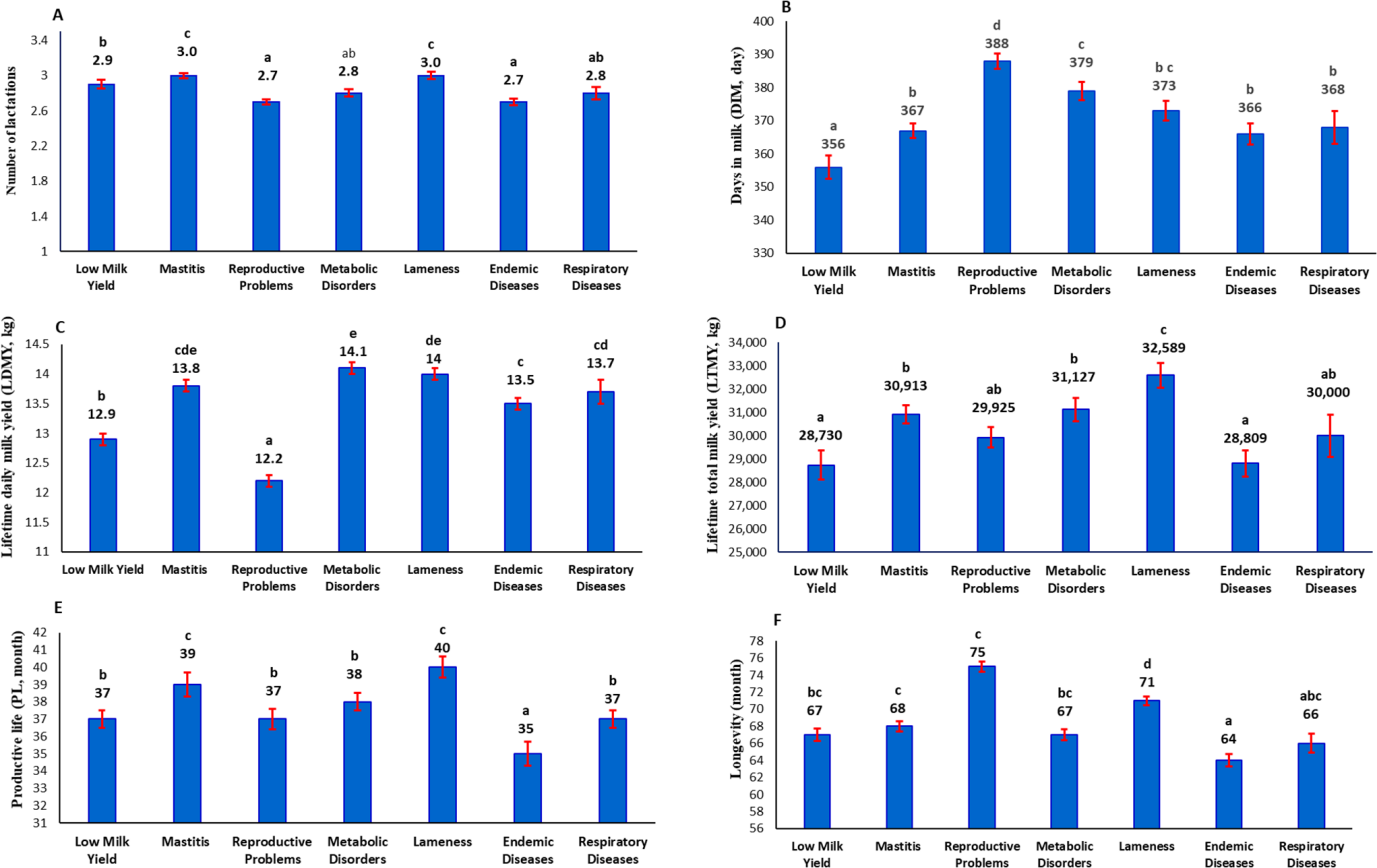
Effects of culling causes on number of lactations (**A**), days in milk (**B**), lifetime daily milk yield (**C**), lifetime total milk yield (**D**), productive life (**E**), and longevity (**F**) in Holstein cows. Bars with different superscript letters (a, b, c, d, e) are significantly different (*p* < 0.05)

Causes of culling influenced the productive traits (*p* < 0.0001). Figure 1A presented that cows culled due to mastitis or lameness recorded the highest NL, while those culled due to reproductive problems or endemic diseases showed the lowest.

As shown in Fig. 1B, the longest DIM was scored by cows culled due to reproductive problems, followed by those with metabolic disorders. Cows culled for lameness showed comparatively long DIM. In contrast, cows culled with low milk yield recorded the shortest DIM.

Figure 1C showed that cows culled that had reproductive problems displayed the lowest LDMY. In contrast, cows culled due to metabolic disorders or lameness scored the highest.

As presented in Fig. 1D, cows culled due to lameness recorded the greatest LTMY, followed by those culled for metabolic disorders or mastitis. The lowest LTMY was found in cows culled due to low milk yield or endemic diseases.

Figure 1E and 1F displayed that cows had reproductive problems, recorded the best longevity at 75 months, despite recording a relatively short PL at 37 months. Cows culled due to endemic diseases had the shortest PL and longevity.

#### Reproductive traits

**Fig. 2:**
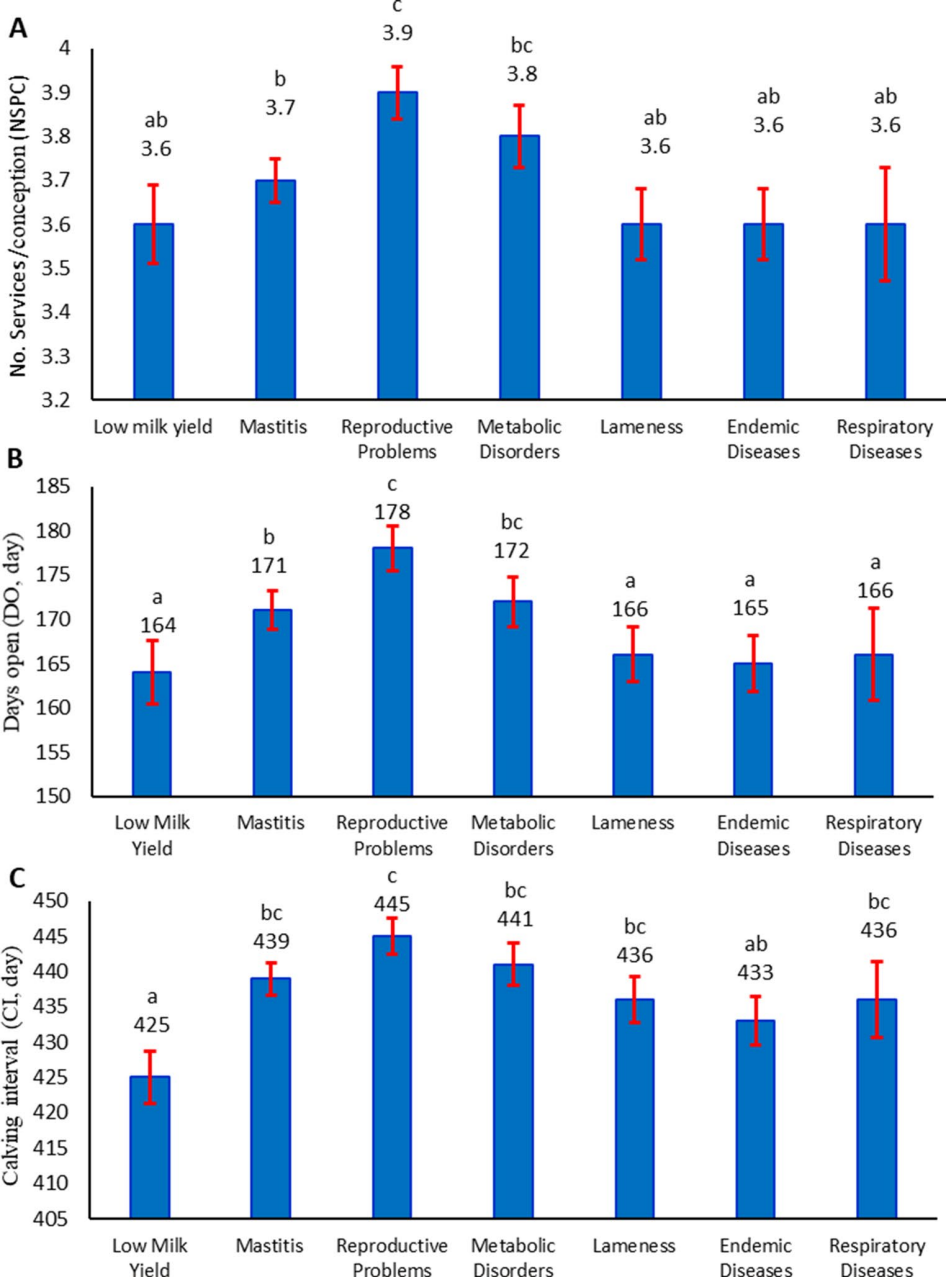
Effects of culling causes on no. services/conception (**A**), days open (**B**), and calving interval (**C**) in Holstein cows. Bars with different superscript letters (a, b, c,) are significantly different (*p* < 0.05)

Culling causes significantly influenced the reproductive traits (*p* < 0.0001). Figure 2A exhibited that cows culled due to reproductive problems recorded the highest NSPC, followed closely by those removed for metabolic disorders. Mastitis was linked with a slightly high NSPC. Conversely, cows culled for low milk yield, lameness, endemic, or respiratory diseases exhibited lower NSPC values.

Figure 2B and 2C showed that cows culled due to reproductive problems displayed the poorest reproductive performance, with the highest values for DO and CI. Similarly, cows culled for metabolic disorders also showed extended periods. There were no significant differences between the two cows’ categories. Conversely, cows culled for lameness, endemic, or respiratory diseases recorded shorter DO and CI than the overall means. The shortest DO and CI were observed in cows culled for low milk yield.

## Discussion

The performance traits of the examined cows in this study were generally in line with findings from previous research on the same breed raised in various countries. The average NL was consistent with values reported in the USA (Hare et al. 2006) and Serbia (Kučević et al. 2020). Similarly, DIM fell within the range observed in Egypt (Gabr 2005; Ibrahim 2006; Farrag et al. 2017), and PL was comparable to reports from Serbia (Sawa and Bogucki 2017) and Poland (Kučević et al. 2020). The observed average longevity aligned with the 4.5–6.6 years range reported by Cielava et al. (2017), while LTMY also fell within the range stated by Cielava et al. (2017) and Kučević et al. (2020).

On the contrary, reproductive performance can only be compared with the averages obtained from Egyptian studies (Gabr 2005; Ibrahim 2006; Osman et al. 2013; Farrag et al. 2017). This result may be returned to the semi-arid climatic conditions prevailing in Egypt, in which cows are exposed to heat for a long time, leading to hormonal disruption due to altered hypothalamic-pituitary-ovarian axis function, reducing estrus expression and follicle development (Penev et al. 2021). It also leads to elevated body temperatures, extending calving intervals, and lowering conception rates (Rhoads 2023). The conception rate of cows raised under severe heat may drop from 63% to 43.5% (Bouamra et al. 2024). In addition, the extended days open and more services per conception increase production costs (Mohammed et al. 2024; Penev et al. 2021). Generally, individual genetics, production systems, ambient conditions, feeds, diseases, and management practices can all contribute to variations within performance trait averages.

### Impacts of first-lactation milk yield (FMY) on lifetime performance traits

The PL and LTMY longevity increased as the FMY increased, consistent with Sawa and Krȩzel-Czopek (2009). FMY has been suggested as a working selection trait in dairy cows. However, the increase in FMY was associated with decreased reproductive performance, as higher FMY was associated with a prolonged interval to first service postpartum, leading to extended DO and a longer CI. In addition, cows with higher FMY needed more NSPC and showed lower conception rates, resulting in reduced NL. Knop and Cernescu (2009) and Riecka and Candrák (2011) have shown the antagonistic link between milk production and reproduction. Cows that produced over 12,000 kg of FMY recorded the longest DIM and the highest LTMY. Consequently, these cows also showed the greatest averages of PL and longevity, lowering their risk of early culling. Applying an extended lactation strategy may enhance milk yield and improve longevity in dairy cows, as also supported by Sehested et al. (2019).

### Impacts of culling causes on lifetime performance traits

#### Productive traits

Cows culled due to mastitis scored the highest PL, which can be attributed to the lengthy period these cows stayed in the herd, as mastitis severity increases with advances in age, accompanied by increased milk production (Abdu and Yusuf 2018). The high LTMY and LDMY displayed by these cows assured that high-yielding cows were more exposed to mastitis, as Tian et al. (2024) stated. Because high-yielding cows produce more milk, the teat canal usually stays open for extended periods, increasing the risk of mastitis-causing bacteria invasion (Singha et al. 2021). Besides, these cows may be milked more times than low-yielders and thus are more exposed to the pressure of the milking machine and the chemicals used through the milking routine, which may affect the teat wall, causing some cracks that may allow more penetration of the pathogens of mastitis. In this study, mastitis was the primary reason for culling, indicating that many high-yielding cows were lost. These lost numbers could be minimized by following udder health hygiene, operating milking machines efficiently, maintaining barns clean and dry, separating the infected animals, using proper identification and treatment, and applying dry cow antibiotic therapy protocol.

Furthermore, cows had reproductive problems recorded the highest longevity (75 months). However, this was not accompanied by the longest PL, 37 months, meaning these cows spent nearly half their lives unproductively. This long, unproductive life explained these cows’ low lifetime performance traits. They produced the lowest LDMY of 12.2 kg among cows culled for other reasons, and their LTMY (29,925 kg) was below the herd average (30,223 kg). These outcomes highlight the problems of keeping cows with reproductive problems. Dairy managers may consider extending the lactation period to compensate for poor reproductive performance. Long-term data suggest that this strategy leads to economic losses. As a result, optimizing nutrition and appropriate management for improved reproductive performance is crucial. Like milk production, it should also receive more attention in breeding programs.

Moreover, cows culled for metabolic disorders achieved the highest LDMY (14.1 kg). It is well established that high-yielding cows are more exposed to metabolic problems (Bollwein et al. 2014). High milk yield necessitates a high metabolic rate to provide the cow with the energy required for milk synthesis. Insufficient feed intake causes a negative energy balance, resulting in the mobilization of bodily stores for energy (Wang et al. 2022). Then, cows show altered milk metabolite profiles, with increased concentrations of specific metabolites like citrate and creatinine, indicating metabolic stress (Xu et al. 2018, 2020). The incidence of metabolic diseases like ketosis and acidosis may impair overall health and productivity (Gross and Bruckmaier 2019; Kapusniaková 2023). Appropriate management and nutritional interventions can alleviate the adverse effects on milk yield.

In addition, cows culled due to lameness had the highest LTMY (32,589 kg), which may be attributed to their longer PL and higher LDMY than the herd averages. Their increased longevity is likely because lame cows are typically culled at more advanced ages (Olechnowicz and Jaskowski 2011). The culls due to lameness were associated with parity order, as reported by Fahim et al. (2021), who used the same data in the current study. This outcome was consistent with Oehm et al. (2019). Older cows have increased odds of lameness, with odds ratios of 3.92 for parity 3 and 8.60 for parity ≤ 4 (Somers et al. 2019). The high culls due to lameness in old age may be attributed to the cow’s increasing weight with age, causing a heaviness on the feet. Likewise, their milk production for a longer number of lactations may lead to the withdrawal of essential nutrients and minerals from the body, which may affect the health of the feet.

Additionally, cows culled for endemic diseases exhibited the shortest longevity and PL; as a result, they recorded the lowest LTMY (28,809 kg). This low performance was because endemic viral diseases in the data of this study led to culling after the first parity (Fahim et al. 2021), preventing cows from reaching their peak output in the third and fourth lactations (Bogucki 2017). This result was consistent with Refaei et al. (2020), who stated that primiparous cows were more exposed to foot and mouth disease due to higher spread infection rates and virus loads during lactation. In addition, the influence of bovine ephemeral fever in first parity cows is crucial since these cows form the basis for herd sustainability (Yeruham et al. 2005).

#### Reproductive traits

Cows culled due to low milk yield showed a high reproductive performance, represented by the lowest DO and CI among all cows. This can be attributed to the antagonistic link between milk production and reproduction (Pires et al. 2015). Low-yielders had less stress than high-yielders. Low insulin, high prolactin, and somatotropin levels are linked to increased milk supply, especially during early lactation (Staufenbiel et al. 1992; Menzies et al. 2009). These concentrations may adversely affect reproduction (Mirzaie et al. 2022). Besides, the negative energy balance resulting from trying to meet the requirements of high-yielding cows may influence hypothalamic GnRH secretion and its subsequent impact on gonadotropin secretion (Nebel and McGilliard 1993), causing a delay in the formation of oocytes, estrus, and ovulation.

Furthermore, cows culled for metabolic diseases showed high NSPC, DO, and CI, with no significant differences from those for reproductive problems. The decline in reproductive performance was not limited to cows with obvious reproductive issues. This suggests that a failure to feed cows according to their physiological status or production may have led to metabolic disorders that impaired fertility. Farman et al. (2016) found that negative energy balance during the first week postpartum impairs ovarian activity in high-yielders, mainly by disrupting follicle growth and hormone production. Elevated levels of non-esterified fatty acids and urea accumulate in dominant follicles, hindering oocyte maturation and reducing fertilization and embryo development success. Besides, research has demonstrated the connection between uterine involution delays and metabolic problems such as lipolysis, hyperketonemia, and hypocalcemia (Bisinotto et al. 2011). These disorders were associated with high NSPC and delays in the return to cyclicity (Paiano et al. 2019), extending CI and thus DO. Furthermore, abnormalities in the composition of follicular fluid could arise in cows with blood metabolic disorders, affecting oocyte development, follicular steroidogenesis, cyclical resumption, and fertility (Leroy et al. 2008; Ribeiro et al. 2013).

The cows culled for mastitis also displayed low reproductive performance. Since mastitis and reproduction are both complex, it is difficult to assess how they relate fully; thus, various mechanisms have been proposed (Kumar et al. 2017). One mechanism was that mastitis raises body temperature; high temperatures can suppress progesterone secretion by luteal cells, preventing granulosa cells from synthesizing steroids, disrupting oocyte and embryonic development (Wolfenson et al. 2000). Another pathway involved immune reactions started by infection of the mammary gland, causing abnormal creation of cytokines and hormones, affecting the ovary, corpus luteum, and hypothalamic-pituitary-gonadal axis causing imbalances in hormonal secretions, including as progesterone, oestrogen, LH, FSH, and GnRH led to aberrant follicular growth, delayed estrus, and increased apoptosis in the embryo (Wang et al. 2021).

Metabolic disorders and mastitis may interfere with reproduction, resulting in reproductive issues, which may lead to cow disposal. The cause of disposal will be documented as a reproductive failure, providing false information and diverting our attention from the actual cause of the culling. These findings highlight the necessity of investigating interrelated links among disorders causing culling, since one disorder may contribute to or cause the onset of another.

Cows culled due to lameness, endemic diseases, or respiratory disorders showed better reproductive performance than cows culled for other involuntary causes. This is a good outcome as these three reasons were responsible for over one-quarter of all culling cases in the current study. Minimizing the number of lameness culls could be achieved by paying attention to barn cleanliness and dryness through manure removal at frequent intervals and hoof hygiene. Reducing endemic and respiratory diseases could be realized by the obligation to vaccinate against these diseases on time and by eliminating the means of spreading these diseases.

## Conclusion

This study underlines the significant effect of first-lactation milk yield and culling causes in shaping the lifetime performance of Holstein cows. Higher first-lactation yields were associated with superior production, but were connected to compromised reproduction. Moreover, lifetime performance differed obviously by culling cause. Cows culled for lameness or metabolic disorders attained the highest lifetime milk yield. Reproductive problems were particularly detrimental, leading to extended longevity but limited productivity. These findings highlight the necessity of integrating early lactation performance and health-related culling risks into herd management and breeding programs. Additionally, these programs should focus on the interrelationships among culling causes, as multiple disorders may overlap or trigger one another, influencing productivity and survival.

## Data Availability

The data sheets are available from the corresponding author if the editor requests them from the journal while publishing the current paper.
